# ^68^Ga-DOTATOC PET/CT to detect immune checkpoint inhibitor-related myocarditis

**DOI:** 10.1136/jitc-2021-003594

**Published:** 2021-10-21

**Authors:** Sarah Boughdad, Sofiya Latifyan, Craig Fenwick, Hasna Bouchaab, Madeleine Suffiotti, Javid J Moslehi, Joe-Elie Salem, Niklaus Schaefer, Marie Nicod-Lalonde, Julien Costes, Matthieu Perreau, Olivier Michielin, Solange Peters, John O Prior, Michel Obeid

**Affiliations:** 1 Nuclear Medicine and Molecular Imaging, Lausanne University Hospital, Lausanne, Switzerland; 2 Department of Medical Oncology, Lausanne University Hospital, Lausanne, Switzerland; 3 Immunology and allergy division, Department of Medicine, Lausanne University Hospital, Lausanne, Switzerland; 4 Section of Cardio-Oncology & Immunology, Division of Cardiology and the Cardiovascular Research Institute, University of California San Francisco, San Francisco, California, USA; 5 Cardio-Oncology Program, Pitié-Salpétrière Hospital, Paris, France, AP-HP, Pitié-Salpétrière Hospital,Sorbonne, INSERM, CIC-1901, Paris, France, Paris, France, France; 6 Radiopharmacy Unit, Department of Pharmacy, Lausanne University Hospital, Lausanne, Switzerland

**Keywords:** immunotherapy

## Abstract

**Background:**

Immune checkpoint inhibitor (ICI)-related myocarditis is a rare but potentially fatal adverse event that can occur following ICI exposure. Early diagnosis and treatment are key to improve patient outcomes. Somatostatin receptor-based positron emission tomography–CT (PET/CT) showed promising results for the assessment of myocardial inflammation, yet information regarding its value for the diagnosis of ICI-related myocarditis, especially at the early stage, is limited. Thus, we investigated the value of ^68^Ga-DOTA(0)-Phe(1)-Tyr(3)-octreotide (^68^Ga-DOTATOC) PET/CT for the early detection and diagnosis of ICI-related myocarditis.

**Methods:**

Consecutive patients with clinically suspected ICI-related myocarditis from July 2018 to February 2021 were retrospectively evaluated in this single-center study. All patients underwent imaging for the detection of ICI-related myocarditis using either cardiac magnetic resonance (CMR) imaging or ^68^Ga-DOTATOC PET/CT. PET/CT images were acquired 90 min after the injection of 2 MBq/kg ^68^Ga-DOTATOC with pathological myocardial uptake in the left ventricle (LV) suggestive of myocarditis defined using a myocardium-to-background ratio of peak standard uptake value to mean intracavitary LV standard uptake (MBR_peak_) value above 1.6. Patients had a full cardiological work-up including ECG, echocardiography, serum cardiac troponin I (cTnI), cardiac troponin T and creatine kinase (CK), CK-MB. Endomyocardial biopsy and inflammatory cytokine markers were also analyzed. The detection rate of ICI-related myocarditis using ^68^Ga-DOTATOC PET/CT and CMR was assessed.

**Results:**

A total of 11 patients had clinically suspected ICI-related myocarditis; 9 underwent ^68^Ga -DOTATOC PET/CT. All nine (100%) patients with ^68^Ga-DOTATOC PET/CT presented with pathological myocardial uptake in the LV that was suggestive of myocarditis (MBR_peak_ of 3.2±0.8, range 2.2–4.4). Eight patients had CMR imaging and 3/8 (38%) patients had lesions evocative of myocarditis. All PET-positive patients were previously treated with a high dose of steroids and intravenous immunoglobulin prior to PET/CT had elevated serum cTnI except for one patient for whom PET/CT was delayed several days. Interestingly, in 5/6 (83%) patients who presented with concomitant myositis, pathological uptake was seen on whole-body ^68^Ga-DOTATOC PET/CT images in the skeletal muscles, suggesting an additional advantage of this method to assess the full extent of the disease. In contrast, four patients with CMR imaging had negative findings despite having elevated serum cTnI levels (range 20.5–5896.1 ng/mL), thus defining possible myocarditis. Newly identified immune correlates could provide specific biomarkers for the diagnosis of ICI-related myocarditis. Most tested patients (six of seven patients) had serum increases in the inflammatory cytokine interleukin (IL)-6 and in the chemokines CXCL9, CXCL10, and CXCL13, and the mass cytometry phenotypes of immune cell populations in the blood also showed correlations with myocardial inflammation. Four of five patients with myocarditis exhibited a Th1/Th2 imbalance favoring a pronounced inflammatory Th1, Th1/Th17, and Th17 CD4 memory T-cell response. The high proportion of non-classical monocytes and significantly reduced levels of CD31 in four to five patients was also consistent with an inflammatory disease.

**Conclusion:**

The use of ^68^Ga-DOTATOC PET/CT along with immune correlates is a highly sensitive method to detect ICI-related myocarditis especially in the early stage of myocardial inflammation, as patients with elevated cTnI may present normal CMR imaging results. ^68^Ga-DOTATOC PET/CT is also useful for detecting concomitant myositis. These results need to be confirmed in a larger population of patients and validated against a histological gold standard if available.

## Introduction

Immune checkpoint inhibitors (ICIs), PD-1, PD-L1 and CTLA-4 have been approved in a wide variety of cancers; hence, the optimal and timely management of immune-related adverse events (irAEs), especially life-threatening toxicities, is required.^1–3^ ICI-related myocarditis is a rare but potentially lethal irAE with an incidence of 0.5% to 1.0% but a high mortality rate of up to 60%.^4 5^ The pathophysiology of ICI-related myocarditis has not been clearly elicited, but postmortem assessments of fatal cases have confirmed myocarditis with substantial inflammatory cell infiltrate characterized by the presence of both CD4-positive and CD8-positive T cells and macrophages but not B cells.^6^ These findings suggest that the uncontrolled activation of autoreactive T cells may occur in patients with ICI-related myocarditis. Interestingly, cardiomyocyte PD-L1 expression is upregulated in cardiac injury and inflammation, suggesting that PD-L1 signaling might have a cardioprotective immunomodulatory effect.^7^ Early detection, timely intervention, and the initiation of adequate immunosuppressive (IS) treatment, can reduce mortality and improve the prognosis of ICI-related myocarditis. The measurement of troponin I serum levels has also yielded promising results for the early diagnosis of ICI-associated myocarditis, with a specificity and predictive value of 95% and 62%, respectively, for cardiac involvement in patients with myositis.^8–10^

The diagnosis of myocarditis is complex and is based on the clinical picture and the results of ECG, transthoracic echocardiography, cardiac biomarkers, and exclusion of acute coronary syndrome using coronary angiography. The gold standard for definite myocarditis remains the endomyocardial biopsy (EMB) but the value of cardiac magnetic resonance (CMR) for a non-invasive diagnosis of myocarditis and monitoring disease progression is now recognized.^11^ The CMR features indicating myocarditis are the combination of myocardial edema (using T2-weighted imaging), myocardial hyperemia, and myocardial necrosis/fibrosis following a non-ischemic distribution (using late-gadolinium enhancement (LGE) imaging according to the initial Lake Louise criteria.^12^ Tissue characterization using parametric imaging (T1 mapping, extracellular volume calculation (ECV), and T2 mapping) allowed further improvement of the diagnostic accuracy of CMR by combining the parameters of myocardial edema (T2-weighted imaging or T2 mapping) and non-ischemic injury (LGE, ECV, or T1 mapping).^13 14^ Imaging also plays an important role in the detection of ICI-related myocarditis, although currently supported by less evidence.^15^ Interestingly, in a series of 103 patients with ICI-related myocarditis assessed with CMR, LGE was observed in only 48%, and T2-weighted imaging was positive in only 28%, indicating a lower than expected sensitivity of CMR, especially when CMR was performed early (<4 days) after admission. Furthermore, neither LGE nor T2-weighted imaging was associated with major adverse cardiac events (MACEs).^16^ On the other hand, using modern parametric imaging in a cohort of 136 patients with ICI-related myocarditis, Thavendiranathan *et al* reported that the criteria of non-ischemic injury was present in 95% and the criteria of myocardial edema in 53% of the patients. Increased T1 values alone were found in 78% of myocarditis cases and had an excellent discriminatory value for subsequent major cardiac events.^17^ Myocardial tissue characterization therefore appears as a critical step in the assessment of ICI-related myocarditis: in a series of 35 patients with ICI-related myocarditis, 46% of the patients experienced MACE in the follow-up and the majority of them had normal left ventricle (LV) ejection fraction at admission, highlighting the fact that an initial normal LV function alone cannot exclude the progression toward major cardiac complications.^18^

Molecular imaging could be a valuable addition when the diagnosis of ICI-related myocarditis remains possible but not definite myocarditis after a complete cardiac work-up, as we previously reported in a patient with refractory, severe ICI-associated myocarditis.^2^ Bonaca *et al* proposed a definition of myocarditis for application in clinical trials in the setting of cancer therapeutics, and they included ^18^F-fluorodeoxyglucose (FDG) positron emission tomography–CT (PET/CT) imaging as a potential tool to define probable myocarditis.^19^ However, it is also known that somatostatin receptor is highly expressed by inflammatory cells.^20^ Somatostatin receptor-based PET/CT such as the ^68^Ga-labeled somatostatin analog ^68^Ga-DOTA(0)-Phe(1)-Tyr(3)-octreotide (^68^Ga-DOTATOC) PET/CT also showed interesting results for the assessment of myocardium inflammation with a close spatial correlation with CMR in a study by Lapa *et al*.^20^ In this context, we present our results showing that imaging with ^68^Ga-DOTATOC PET/CT, and the correlation with some specific cytokine correlates might be beneficial in the diagnostic work-up of ICI-related myocarditis.

## Methods

### Patient population and diagnostic work-up

Consecutive patients with clinically suspected ICI-associated myocarditis from July 2018 to February 2021 at our institution were included in this study. All patients underwent a cardiological work-up including cardiac biomarkers (serum cardiac troponin I (cTnI), cardiac troponin T, creatine kinase (CK), CK-MB and inflammatory cytokine markers when available), 12-lead ECG, transthoracic echocardiography (TTE). Most patients underwent CMR imaging for the detection of ICI-associated myocarditis. The results of EMB and coronary angiography were also part of the diagnostic work-up, when available. Based on this assessment, the probability of ICI-related myocarditis was defined according to the recommendations of Bonaca *et al* as definite, probable or possible.

### Echocardiographic and CMR imaging protocol

Complete echocardiographic imaging was performed using either a Vivid E9 (GE Healthcare) or an Epic V.7 (Philips Healthcare) according to the current guidelines.^21^ Biplane Simpson method was used to calculate LV volumes, and the threshold for normal LV ejection fraction was ≥52% in men and ≥54% in women. The presence of regional wall motion abnormalities was specifically assessed by experienced board-certified cardiologists. ECG-gated CMR was performed on a 1.5 T MAGNETOM Sola scanner (Siemens Healthcare) with a 32-channel phased-array surface receiver coil. Imaging protocol for suspected myocarditis included cine images acquired with breath-hold steady-state free precession sequences in short-axis orientation from base to apex (slice thickness 8 mm, no gap) and in three long axis orientation (two-chamber, three-chamber and four-chamber orientations). Edema detection was performed using T2 mapping acquisitions in short-axis orientation from the base to the apex of the LV. Normal values for T2 relaxation time were defined on a segment basis in our center and edema was considered present when the myocardial T2 relaxation time was superior to 2 SDs above the local normal values. T1 mapping sequences were performed both before and after contrast injection, but they were acquired only on a single basal short-axis slice orientation. Ten minutes after the administration of a 0.2 mmol/kg intravenous bolus of Gadobutrol (Gadovist, Bayer Healthcare), LGE images were acquired using a two-dimensional breath-hold phase-sensitive segmented inversion–recovery gradient echo pulse sequence in the same orientations as the cine images. Experienced (European Association of Cardiovascular Imaging level 3) cardiologists visually assessed the presence and distribution of myocardial lesions on LGE images.

### Metabolic imaging with ^68^Ga -DOTATOC PET/CT

^68^Ga-DOTATOC PET/CT was obtained from Biograph Vision Siemens (Erlangen, Germany). Thirty-nine patients with neuroendocrine tumors that underwent ^68^Ga-DOTATOC PET/CT in a diagnosis or follow-up setting without pathological uptake on visual analysis were used as a control group. Pathological uptake in the myocardium of different regions of the LV, namely, the free wall, septum and apex, was calculated and measured with a previously defined threshold of 1.6 for the ratio between myocardium standardized uptake value (SUV) and the average left ventricular SUV (myocardium-to-background ratio or myocardium-to-background ratio of peak standard uptake value to mean intracavitary LV standard uptake (MBR_peak_)), in agreement with the literature and our previous work in the field.^22^ On visual analysis, we described a pathological uptake of ^68^Ga-DOTATOC seen on the three regions of the myocardium (apex, septum, and free wall) as ‘patchy diffuse’, whereas a less extensive pathological uptake was described as ‘heterogeneous’. We compared ratio values calculated on whole body ^68^Ga-DOTATOC PET/CT acquisitions between patients with clinically suspected ICI-associated myocarditis and the control group using non-parametric Mann-Withney test and performed a receiver operating characteristic (ROC) analysis for determining the presence of a myocarditis.^19^

### Cytokine panel

To define the specific serum cytokine signature associated with ICI-related myocarditis, the concentration of serum markers in different patients was evaluated by a multiplex bead assay (Thermo Fisher) at different time points before and after tocilizumab (TCZ) treatment. Briefly, serum concentrations of cytokines and soluble cytokine receptors (eg, IL-1alpha, IL-1RA, IL-1beta, IL-2, IL-4, IL-5, IL-6, IL-7, IL-9, IL-10, IL-12p70, IL-13, IL-15, IL-17A, IL-18, IL-21, IL −22, IL-23, IL-27, IL-31, interferon (IFN)-α, IFN-γ, and tumor necrosis factor (TNF)-α), chemokines (eg, CCL2 (MCP-1), CCL3 (MIP-1α), CCL4 (MIP-1β), CCL5 (RANTES), CCL11 (Eotaxin), CXCL1 (GRO-α/KC), CXCL8 (IL-8), CXCL9 (MIG), CXCL10 (IP-10), CXCL12 (SDF-1α), CXCL13 (BLC), and TNF-beta), and growth factors (eg, NGF-beta, BDNF, EGF, FGF-2, HGF, LIF, PDGF-BB, PlGF-1, SCF, VEGF-A, VEGF-D, BAFF, GM-CSF, and G-CSF) were determined by multiplex bead assays as previously described.^23^ The reference values of these 49 serum markers were defined on the basis of the results obtained from 450 healthy subjects. The references are age-adjusted values and validated by the Centre Hospitalier Universitaire Vaudois (CHUV) Immunology and Allergy diagnostics platforms for clinical routine practice. ROC curves were generated to evaluate the discriminative ability of each cytokine to separate patients with ICI-related myocarditis from controls. The areas under the ROC curves (AUC values), sensitivity, specificity, positive predictive value (ppv), negative predictive value (npv) for cytokines of interest were calculated. In addition, principal component analysis (PCA) was performed to evaluate the variability and to determine which markers mostly contribute to the separation of control and ICI-related myocarditis groups. All calculations were performed using R software V.3.6.3 and prcomp function for PCA analysis.

### Mass cytometry (CyTOF)

As described previously,^24 25^ fresh blood samples were mixed with 1× red blood cell lysis buffer (eBiosciences) to remove erythrocytes according to the manufacturer’s protocol. Pelleted cells were stained using metal-conjugated antibodies according to the CyTOF manufacturer’s instructions (Fluidigm, San Francisco, California, USA). Through direct staining in whole blood, patient immune cell distributions will be compared with age-adjusted values for 450 normal healthy donors and longitudinal changes were monitored over the course of the ICI therapy. The CyTOF panel will consist of 28 markers for cell surface staining and up to 12 intracellular markers to evaluate the accumulation of intracellular cytokines and cytotoxic molecules. The panel included conjugated antibodies against CD45 141 Pr, CD3 154 Sm, CD4 115 In, CD8 115 In, CD19 142 Nd, CD27 155 Gd, CCR7 159 Tb, CD14 160 Gd, CD11c 162 Dy, CXCR3 163 Dy, CD38 167 Er, CD25 169 Tm, HLA-DR 174 Yb, PD-1 175 Lu, and CD16 209 Bi (Fluidigm. Cells were subsequently washed with cell staining medium and phosphate-buffered saline (PBS), fixed with 2.4% formaldehyde (Thermo Fisher) in PBS for 5 min and then resuspended in DNA intercalation solution (PBS, 1 µM Ir-Intercalator, 1% formaldehyde, 0.3% saponin) and stored at 4°C until analysis. For CyTOF analysis, cells were washed three times with Milli-Q water and resuspended at 0.5×10E6 cells/mL in 0.1% EQ Four Element Calibration Beads solution (Fluidigm). Samples were acquired on a Helios instrument at a flow rate of 30 µL/min. Flow Cytometry Standard files were normalized for the EQ bead intensities using MATLAB normalizer software to limit interanalysis staining intensities. Data were processed and analyzed with the Cytobank software package. The references are age-adjusted values and validated by the CHUV Immunology and Allergy diagnostics platforms for clinical routine practice.

## Results

### Patient characteristics

Eleven consecutive patients (1 woman and 10 men) with various cancers treated with ICIs were admitted to the hospital for suspected ICI-related myocarditis. Nine of them had a ^68^Ga-DOTATOC PET/CT performed in the diagnostic work-up and were included in the study (online supplemental figure S1A). The clinical characteristics of the patients are presented in table 1. The mean age of the patients (n=11) who developed ICI-associated myocarditis was 71 years±11 (table 1). Before myocarditis, the pre-ICI left ventricular ejection fraction (LVEF) was normal, ≥52% in men and ≥54% in women in 10 out of the 11 patients with a baseline measurement. Six patients out of 11 (45%) received ICIs in the setting of melanoma, and 2 patients were on their second line of ICIs when ICI-related myocarditis was diagnosed. Six patients (54%) received a combination of the ICIs ipilimumab–nivolumab. Overall, myocarditis occurred more commonly (54%) in patients receiving a combination of ICIs at the time of presentation. All patients had experienced at least another ICI-related side effect: myositis (80%, 8 of 11), including 2 with ocular myositis without myasthenia gravis, hepatitis (18%) and colitis (18%) (table 1). For all the patients included in this study, ICI treatment was interrupted once the diagnosis of ICI-associated myocarditis was suspected, and appropriate treatment with steroids and additional IS drugs was initiated in the case of corticosteroid (CS)-refractory myocarditis.

10.1136/jitc-2021-003594.supp1 Supplementary data

10.1136/jitc-2021-003594.supp2 Supplementary data

**Figure 1:**
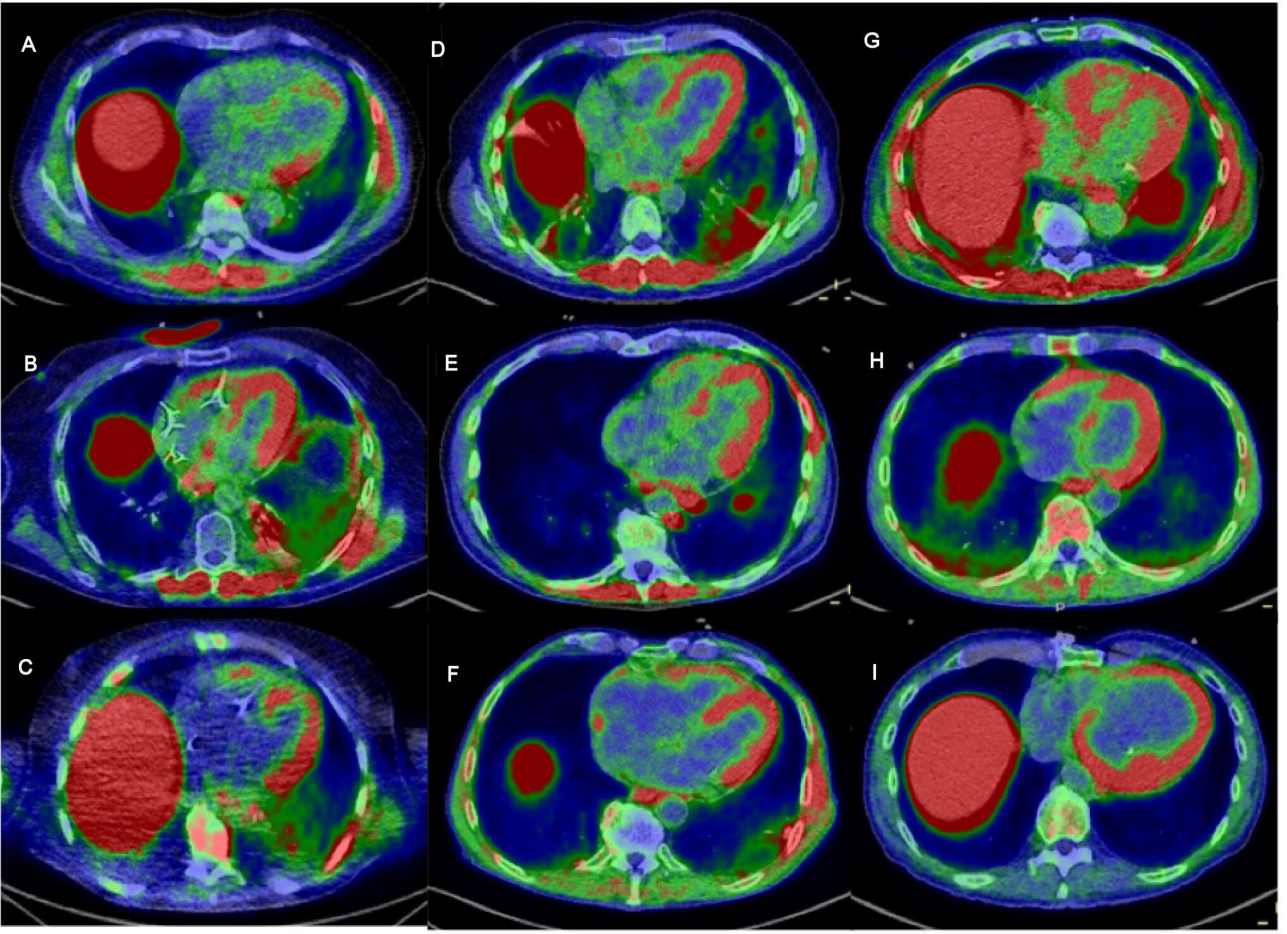
Fused ^68^Ga -DOTATOC PET/CT images in the axial plane centered on the long axis of the LV (A–I): patients 3–11 with pathological uptake in the myocardium; pathological uptake in the paravertebral and intercostal muscle is also seen in some patients (A, B, D, E, G); SUV scale 0–2 g/mL. ^68^Ga -DOTATOC, ^68^Ga-DOTA(0)-Phe(1)-Tyr(3)-octreotide; LV, left ventricle.

**Table 1:** Clinical characteristics of the patients

Patient ID	Age/gender	Cancer	Previous chemotherapy	TNM cancer staging	ICI (number of cycles)	irAEs (grade)	Treatments	Clinical resolution
Patient 1	77/F	Merkel carcinoma	No	Stade IIA	Pembrolizumab (six cycles)	Myocarditis G2 Pericarditis	None	Yes
Patient 2	79.6/M	Melanoma	No	Stage IV	First line: pembrolizumab (two cycles) Second line: ipilimumab and nivolumab (three cycles)	Myocarditis G3 Ocular myositis	CS+infliximab	Yes
Patient 3	64.9/M	Melanoma	No	Stage IIIC	Pembrolizumab (two cycles)	Myocarditis (G3) Hepatitis (G3) Myositis (G2)	CS+MMF+infliximab	Yes
Patient 4	57.7/M	Small cell neuro-endocrine carcinoma	Cisplatine–etoposide	Stage IIIB	Ipilimumab and nivolumab (two cycles)	Myocarditis (G3–G4) Ocular myositis	CS+TCZ	Yes
Patient 5	66.3/M	Prostate adenocarcinoma	Taxotere and cabazitaxel	Stage IV	Nivolumab (one cycle)	Myocarditis (G3–G4) Myositis	CS+MMF+TCZ	Yes
Patient 6	64.9/M	Squamous cell anal carcinoma	Capecitabine–mytomycin	Recurrence (LN	Pembrolizumab (two cycles)	Myocarditis (G3) Hepatitis (G2) Myositis (G3)	CS	Yes
Patient 7	84.6/M	Melanoma	No	Stage IV	Nivolumab (four cycles)	Myocarditis (G2) Myositis	CS	Yes
Patient 8	87.7/M	Melanoma	No	Stage IV	First line: pembrolizumab (two cycles) Second line: ipilimumab and nivolumab (two cycles)	Myocarditis (G3–G4) Myositis	CS+TCZ	Yes
Patient 9	74.9/M	Hepatocellular carcinoma	No	Stage IV	Atezolizumab (four cycles)	Myocarditis (G2) Myositis	CS	Yes
Patient 10	56/M	Melanoma	No	Stage IV	Ipilimumab and nivolumab (four cycles)	Myocarditis (G1) Colitis (G3) Acute interstitial nephritis	CS+infliximab+TCZ	Yes
Patient 11	64.6/M	Melanoma	No	Stage IIIC	First line: nivolumab (16 cycles) Second line: ipilimumab and nivolumab (four cycles)	Myocarditis (G1) Colitis (G3) Thyroiditis Hypophysitis	CS+infliximab	Yes

CS, corticosteroid; F, female; ICI, immune checkpoint inhibitor; irAE, immune-related adverse event; LN, lymph node; M, male; MMF, mycophenolate mofetil; TCZ, tocilizumab; TNM, tumour, node, metastasis.

### Myocarditis presentation

The clinical and biological characteristics of the patients are shown in table 2 (online supplemental figure S1B). The median time to the onset of myocarditis from ICI initiation (or the first day of second-line ICI treatment for three patients) was 63 days (IQR 11–124 days), with 91% presenting within 3 months of therapy initiation. Eight patients presented with cardiac symptoms (mainly chest pain) at diagnosis, and early investigations showed new alterations on ECG in 4 out of 10 patients (4 atrioventricular block and 1 right branch block), whereas no alterations in the left ventricular function were seen on TTE. Indeed only one patient had an altered left ventricular function at diagnosis (18% EF) with a similar ECG to a previous one done 6 months before. All myocarditis (11 out of 11) patients had a high-sensitivity (hs) troponin T elevation (100%), though it was subnormal in one patient, and nearly all patients had an hs troponin I (hs troponin I) elevation (80%, 8 out of 10). Besides, one patient had a cardiac biopsy.

**Table 2:** Imaging and biological characteristics of the patients

Patient ID	^68^Ga-DOTATOC PET/CT			CMR imaging	hs troponine I values (n >25 ng/L)	Other investigations	Classification bonaca *et al*
	Uptake pattern	Highest ratio SUV_peak_M_/SUV_mean_IC_ *	Days from symptom onset and PET/CT				
Patient 1	ND	ND	ND	Intramyocardial lesions, edema and pericarditis	ND	TTE =no alteration of LV function/ECG=no kinetic alterations/hs troponin T=229 ng/L (n <14 ng/L)/creatinin kinase=140 CK U/L (N 25–190) U/L	Definite
Patient 2	ND	ND	ND	Minimal subepicardial lesions, no edema	18.8	TTE=no alteration of LV function/ECG=no kinetic alteration/troponin T=387 ng/L/CK=178 U/L	Definite
Patient 3	Heterogenous	2.2	7	Absence of edema or intramyocardial scars of inflammatory type	88.7	TTE=ND/ECG=AVB I and LAHB/troponin T=1231 ng/L/CK=6249 U/L	Possible
Patient 4	Patchy diffuse	2.3	3	ND	981.9	TTE=no alteration of LV function/ECG=new AVB/troponin T=1045 ng/L (<14) /CK=2234 U/L (<190)	Possible
Patient 5	Patchy diffuse	3.1	4	ND	169.9	TTE=no alteration of LV function or kinetics/ECG=new intermittent high-grade AVB/troponin T=2389 ng/L/CK=524 U/L	Possible
Patient 6	Patchy diffuse	4.4	10 †	Absence of edema or intramyocardial scars of inflammatory type.	1604.7	TTE=no alteration of LV function or kinetics/ECG=diffuse alterations of the repolarization with submillimetre undershift and flattened T wave/troponin T=1281 ng/L/CK=4752 U/L	Possible
Patient 7	Patchy diffuse	2.9	3	Absence of edema or intramyocardial scars of inflammatory type	212.6	TTE=no alteration of LV function or kinetics/ECG=sinusal rythm, no modification/troponin T=936 ng/L/CK=844 U/L	Possible
Patient 8	Patchy diffuse	3.3	5	Late-gadolinium enhancement (patchy pattern) and edema	5896.1	TTE=no alteration of LV function or kinetics/ECG=new bundle branch block/troponin T=966 ng/L/CK=127 U/L	Definite
Patient 9	Heterogenous	2.4	3	Absence of edema or intramyocardial scars of inflammatory type	20.5	TTE=NF/ECG=sinusal rythm/troponin T=1210 ng/L/CK=280 U/L	Possible
Patient 10	Heterogenous	3.5	1 †	Absence of edema or intramyocardial scars of inflammatory type	28	TTE=no alteration of LV function or kinetics, minimal pericardial effusion/ECG=sinusal rythm/troponin T=40 ng/L/CK=235 U/L	Possible
Patient 11	Heterogenous	4.4	13	ND	110	TTE=low LV function (18%) and alterations of segmental kinetics/ECG=sinusal rythm/troponin T=150 ng/L/CK=40 U/L	Probable

Troponin I normal values <26 ng/L; troponin T normal values <14 ng/L; CK normal values <190 ng/L.

*MBR_peak_: SUV_peak_M_ to left ventricular intracavitary background SUV_mean_ ratio.

†Two patients had immunosuppressive treatment initiated on the same day as the PET/CT.

AVB, atrioventricular block; CK, creatine kinase; 68Ga-DOTATOC, ^68^Ga-DOTA(0)-Phe(1)-Tyr(3)-octreotide; hs, high-sensitivity; LAHB, left anterior hemiblock; LV, left ventricle; MBR_peak_, myocardium-to-background ratio of myocardium SUVpeak to LV intracavitary SUVmean; ND, not done; NF, not found; PET, positron emission tomography; SUV_peak_M_, myocardial SUV_peak_; TTE, transthoracic echocardiogram.

### Myocarditis imaging

Among those patients, nine underwent ^68^Ga-DOTATOC PET/CT for the diagnosis of ICI-associated myocarditis, whereas eight underwent CMR imaging. Seven patients had both PET/CT and CMR imaging. All patients except one patient had measurements of hs troponin I serum levels. All patients who underwent PET/CT imaging had pathological uptake in the myocardium compared with the intraventricular chamber, and the most frequent pattern of uptake seen was patchy diffuse uptake in the myocardium (figure 1). For patients who underwent both imaging PET/CT and CRM imaging, the rest of the six patients out of the eight were discordant (table 2). Interestingly, for five patients with negative CMR imaging results but positive PET/CT results, hs troponin I serum levels were elevated (table 2 and online supplemental figure S2). A total of 37 patients (18 women and 19 men) with 39 consecutive scans were included in the control group, with a mean age of 57.4 years (19.1–82.8). Amidst this control population, 17 out of 33 patients (clinical history was not available for 4 patients) had no clinical history of cardiovascular disease or risk factors; 1 patient had a history of myocardial infarction; 3 had a history of cardiovascular disease; and 12 had at least one risk factor. Looking at the nine patients with clinical suspicion of irAEs—myocarditis, one patient had a history of myocardial infarction; two had a history of cardiovascular disease; and five had at least one risk factor. There were significant differences for all the ratio values calculated on different regions of the LV myocardium (free wall, septum, and apex) between the patients with clinically suspected ICI-associated myocarditis and the control group (p value <0.001) with good diagnostic performance on ROC analysis using a value of 2 for the ratio on the free wall (AUC 0.954, 94% sensitivity, and 87% specificity; online supplemental figure S3).

10.1136/jitc-2021-003594.supp3 Supplementary data

10.1136/jitc-2021-003594.supp4 Supplementary data

^68^Ga-DOTATOC PET/CT was performed on an average of 5.4 days (1–13 days) after the onset of clinical symptoms, with pathological uptake detected in 100% of patients with elevated troponin T, 100% of patients with elevated NT-proBNP, 100% of patients with reduced LVEF and 100% of patients with abnormal ECG. CMR was performed an average of 3.0 days±3.1 days after the onset of clinical symptoms, with suggestive myocardial lesions seen in only 20% of the patients with elevated troponin T, 50% of patients with elevated N-terminal pro-brain natriuretic peptide (NT-proBNP), none of patients with reduced LVEF and 50% of patients with abnormal ECG (table 2). According to the recommendations for the definition from Bonaca *et al* (15), we defined 3 patients as definite, 1 as probable and seven as possible in our population (table 2). Looking at PET results, among the 5 patients with patchy diffuse uptake one had a definite myocarditis according to Bonaca *et al* whereas for patient with a probable myocarditis the uptake on ^68^Ga-DOTATOC PET/CT was patchy (table 2). We did not look into a possible association between Bonaca *et al* criteria and MBRpeak as among the 9 patients who had a PET/CT, 7 were defined as possible myocarditis, 1 with definite and the other with probable, thus not allowing a comparison between groups.

Additionally, in five out six patients who presented with concomitant myositis, pathological uptake was seen in the muscles, especially the paravertebral and intercostal musculature, as well as the diaphragm on PET/CT imaging (online supplemental figure S4A). CK levels were also elevated in four of the patients with significant uptake muscle uptake on ^68^Ga-DOTATOC PET/CT. One patient presented with ocular myositis, which was difficult to detect on PET/CT imaging. Significant uptake in the mediastinal and hilar lymph nodes was also seen in several patients, with one patient having very intense uptake, suggesting a highly intense locoregional inflammatory response (online supplemental figure S4B). Additionally, one patient with acute biological autoimmune pancreatitis presented with high and diffuse pancreatic uptake on ^68^Ga-DOTATOC PET/CT (online supplemental figure S4B). Interestingly, some patients with tumorous lesions seen on the ^68^Ga-DOTATOC PET/CT showed moderate uptake, but this was not explored in this study. Conversely, only three out of the eight patients who underwent CMR showed lesions suggestive of ICI-related myocarditis, and no edema or intramyocardial scars of the inflammatory type were seen for the other five patients. CMR did not provide an alternative diagnosis in these patients, particularly ischemic events. Two patients had additional angiography to exclude an ischemic origin, and one patient had a cardiac biopsy that did not show any signs of inflammation in the LV.

10.1136/jitc-2021-003594.supp5 Supplementary data

### Treatments

Of the 11 ICI-related myocarditis cases, only patient one did not have IS treatments. Among patients that had ^68^Ga-DOTATOC PET/CT, seven out of nine patients had IS treatment several days prior to the scan with an average delay of 4.2 days (0–15 days). Two patients had IS treatment initiated on the same day as the PET/CT (table 2). Only three patients (6, 7, and 9) had a satisfactory response to CS without the need for additional IS treatment. Patients 3 and 5 received a third IS regimen with infliximab because of refractoriness to progression with mycophenolate mofetil (MMF) and CS. Two MMF-refractory patients with myocarditis (patients 3 and 5) were treated with infliximab and TCZ, respectively. One patient with infliximab-refractory myocarditis was treated with TCZ. Four patients with CS-refractory myocarditis were treated with infliximab (patients 2 and 11) or TCZ (patients 4 and 8, table 1). All in all, the clinical course for all ICI-related myocarditis were favorable despite CS-refractory cases (table 1) and corroborating with a persistent drop in troponin I following different treatments (online supplemental figure S1B).

### Immune correlates

**Figure 2:**
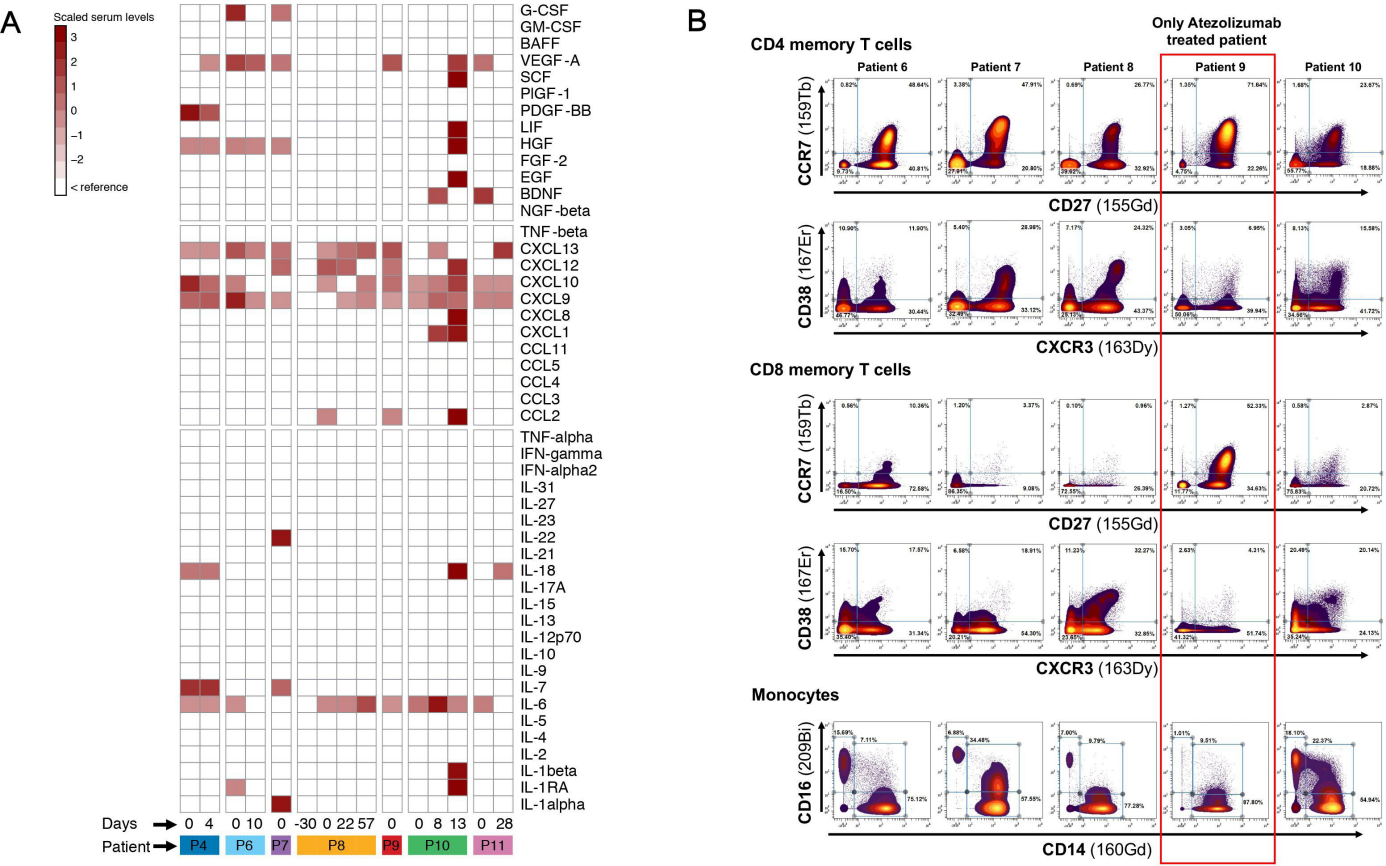
Heatmap of cytokines, chemokines and growth factors identified in patients diagnosed with ICI-related myocarditis. Values are displayed as colors ranging from white to dark red according to deviation to the reference. Day 0 corresponds to the day of clinical presentation of the myocarditis, and the numbers correspond to the delay in days between clinical presentation of the myocarditis and the analysis of the chemokines and cytokines in the serum. ICI, immune checkpoint inhibitor; IFN, tumor necrosis factor; IL, interleukin; TNF, tumor necrosis factor.

Most patients had serum increases in the inflammatory cytokine IL-6 (six of eight patients) and in the chemokines CXCL9, CXCL10 and CXCL13 (five to six of eight patients) (figure 2A). As expected and reported previously, the serum level of IL-6 increased during TCZ treatment.^24 26^ Furthermore, elevated levels of the growth factors hepatocyte growth factor (HGF) and/or vascular endothelial growth factor (VEGF-A) were observed in four of eight patients (figure 2A). Major immune cell populations in blood samples from five patients (patients 6–10) were profiled using a mass cytometry panel consisting of 44 different metal isotope-labeled antibodies targeting immune-related markers for cell lineage, memory subsets, chemokine receptors, and activation markers. Of note, T cells and monocyte immune cell subsets in patient samples showed features that were distinct from those of healthy donor samples. All five patients tested displayed significant increases in the proportion of Th1, Th1/Th17, and Th17 helper memory CD4 T-cell populations that expressed elevated levels of the CXCR3, CXCR3/CCR6, and CCR6/CCR4 chemokine receptors, respectively, compared with a panel of 146 healthy donor controls. Patients also had a corresponding reduction in levels of Th2 helper cells, as defined by CCR4+/CXCR3−/CCR6− expression. Patient memory CD8 T cells also exhibited elevated expression of CXCR3, either with or without the expression of the CD38 activation marker. With regard to the monocyte cell population, the proportion of non-classical monocytes relative to classical and intermediate monocyte populations also increased in four of five patients. Non-classical monocytes from patients also showed significantly lower levels of CD45RA, CD31, and CD1c phenotypical markers than those from healthy donor controls (figure 2B).

ROC curves were constructed showing the abilities of serum cytokines to distinguish patients with ICI-related myocarditis from healthy controls. The diagnostic values of each cytokine, including AUC, cut-off value (value that maximizes sensitivity and minimize false positive rate, sensitivity, specificity, positive predictive value, and negative predictive value are shown in online supplemental figure S5). The cytokines that provided acceptable discrimination for use in the diagnostic test (ie, AUC >0.7) included IL-6, CXCL9, CXCL10, and CXCL13. Among the cytokines with high diagnostic values, IL-6, CXCL9, CXCL10, and CXCL13 had hs (~100 %), while IL-6 and CXCL13 had the lowest specificity (71% and 85%, respectively) in contrast to 100% for CXCL9 and CXCL10 (online supplemental figure S5A). The color distribution of patients with ICI-related myocarditis differed from that of controls. The first principal component (dim 1) accounts for 47.2% of the variability (separation) of control and ICI-related myocarditis groups (online supplemental figure S5B).

10.1136/jitc-2021-003594.supp6 Supplementary data

## Discussion

To the best of our knowledge, this is the first article reporting the usefulnesss of ^68^Ga-DOTATOC for the diagnosis of ICI-related myocarditis at an early stage. Pathological uptake was seen in the myocardium of all the patients with suspected ICI-related myocarditis. In this study, it showed hs for detecting pathological uptake in the myocardium of the LV in patients with clinical symptoms and an increase in troponin levels and inflammatory cytokines that were suggestive of myocarditis. This was confirmed by the comparison of myocardium uptake on whole-body acquisition between our group of patients with clinical suspicion of ICI-related myocarditis and a population of 37 consecutive patients (39 scans) with neuroendocrine tumors that underwent ^68^Ga-DOTATOC PET/CT (online supplemental figure S6). We previously defined a threshold of 1.6 for the MBR_peak_ for the diagnosis of myocarditis on ^68^Ga-DOTATOC PET/CT in a population of 21 patients referred for suspected myocarditis, of whom 14 had pathological uptake in comparison to a control group of 44 patients with neuroendocrine tumors and no clinical history of cardiac inflammation.^22^ However, in routine practice, we tend to favor a higher threshold of 2 for the MBR_peak_ in order to increase specificity and which would have led to similar results in this study (table 2). CMR imaging was negative for six of the eight patients who underwent both imaging modalities, though it should be mentioned that for one patient with altered renal function, CMR was performed without gadolinium contrast, limiting its sensitivity. Moreover, in our opinion, selection bias might have existed, as patients recommended for PET/CT already had a high suspicion of ICI-related myocarditis, with high blood levels of hs troponin I. Indeed, except for one patient, there was a good concordance between a positive PET/CT and elevated blood levels of hs troponin I. The patient who had normal hs troponin I serum levels at the time of PET/CT was treated with a high dose of steroids and intravenous immunoglobulin for several days prior to PET/CT. Additionally, ICI treatment was interrupted, and IS treatment was initiated, at the onset of clinical symptoms, which may have led to a false-negative finding for troponin levels. In addition, this patient underwent a neurological evaluation that confirmed the presence of a motor deficiency in relation to myositis, which was reflected by electromyographic abnormalities.

10.1136/jitc-2021-003594.supp7 Supplementary data

In contrast, among the eight patients with a strong clinical suspicion of ICI-related myocarditis who underwent CMR imaging, its sensitivity for detecting lesions associated with myocarditis was moderate, with a positive result seen in only three patients. These results concord with the recent data in the literature, even though the lack of sensitivity of CMR imaging for the detection of acute myocarditis has been reported.^27^ Interestingly, somatostatin receptors (SSTR)-targeted radiotracers for PET/CT imaging do not present physiological uptake.^28^ The higher sensitivity of ^68^Ga-DOTATOC PET/CT might be related to an infiltration of the myocardium by lymphocytic cells expressing somatostatin receptor 2 (STTR2),^28^ which can be detected before significant myocardial damage detectable by CMR occur. Indeed, SSTR-based PET/CT directly detects myocardium inflammation in vivo, as activated inflammatory cells have been known to overexpress SSTRs (SSTR1 and 2).^28 29^ Borchert *et al* also showed that polarized macrophages, T cells/natural killer (NK) cells and B cells had a higher uptake of ^68^Ga-DOTATATE than neutrophils.^30^ These results are interesting, as myocardial histological findings showed predominantly lymphocytic cells (CD8^+^ and CD4^+^ T cells) with macrophaparge/histiocyte/giant cell (mainly CD68+ cells) cellular infiltration in most cases.^27^ Thus, it could facilitate the detection of myocarditis at an early stage, especially in patients with a diagnosis of possible myocarditis after complete cardiological work-up and for the subsequent therapeutic monitoring.^20 22^ There were no comparisons between ^68^Ga-DOTATOC findings and pathology results, as only one patient (patient 4) had a myocardial biopsy that did not show signs of inflammation despite having highly elevated levels of serum troponin I and inflammatory cytokine markers and a diffuse but heterogeneous pathological uptake in the myocardium on ^68^Ga -DOTATOC PET/CT. EMB is still being considered the gold standard for the definitive diagnosis of myocarditis, but it is technically challenging, which could explain the limited number of patients with pathology data.^29 31 32^

In addition, with ^68^Ga-DOTATOC PET/CT imaging, it is possible to perform whole-body acquisition after a single injection of the radiotracer. In the control group of patients with neuroendocrine tumors, there was no uptake in the muscle (online supplemental figure S6). This allows the detection of concomitant irAEs such as myositis, which is frequently associated with ICI-associated myocarditis or, in one patient, with acute pancreatitis. Another advantage of ^68^Ga-DOTATOC PET/CT imaging is that it is well suited for an emergency setting, as it does not require an extensive carbohydrate-free diet, in contrast to ^18^F-FDG PET/CT.

Immune profiles, as determined by serum levels of cytokines/chemokines and phenotypes of immune cell populations in the blood, also show a correlation with myocardial inflammation. Elevated levels of IL-6 were detected in five of eight patients and are indicative of ongoing inflammation, while increased levels of the CXCL9 and/or CXCL10 chemoattractants in six of eight patients could be involved in the recruitment of CXCR3-expressing immune cells, including cytotoxic lymphocytes, NK cells, and macrophages, to a site of inflammation. Augmented CXCL9 and CXCL10 expression has been shown to be correlated with the development of heart failure, left ventricular dysfunction, and the development of adverse cardiac remodeling.^33 34^

It is quite interesting to note that serum IFN-γ was not increased especially that IFN-γ-induced chemokines such as CXCL9 and CXCL10 and IFN-γ-inducing cytokine such as IL-18 are elevated. Furthermore, CXCL9 production is induced specifically by IFN-γ, but CXCL10 and CXCL11 can also be induced by IFN-α, IFN-β, and TNF-α. In our case series, IFN-α and TNF-α were not increased. Interestingly, it is was reported in both murine models and patients undergoing ICI that macrophages were the predominant source of CXCL9, and their depletion abrogated CD8^+^ T-cell infiltration and the therapeutic efficacy of ICI underlining the fundamental importance of macrophage-derived CXCR3 ligands for therapeutic efficiency of ICI.^35^ This might also account for macrophages infiltrating the myocardium in case of an ICI-related myocarditis because it showed a higher density of CD68+ PD-L1+ macrophages in high-grade ICI-related myocarditis.^36^ Unfortunately, we did not assess the presence of macrophages in myocardial tissue (since there was only one patient biopsied, which was negative). This dissociation between IFN-γ and IFN-γ-induced chemokines was described in other diseases such as patients of polymyalgia rheumatica showed that serum levels CXCL9 and CXCL10 were elevated although the levels of IFN-γ and TNF-α were not increased.^37^ Therefore, it is not well established whether changes in serum chemokines reflect infiltration of immune cells in the myocardium and what is the exact part of macrophage versus CD8^+^ T-cell involvement in the production of the serum CXCL9 and 10.

In line with the cytokine/chemokine profile, four of five patients with myocarditis evaluated by mass cytometry exhibited a Th1/Th2 imbalance that favored a pronounced inflammatory Th1, Th1/Th17 and Th17 CD4 memory T-cell response. The PCA analysis clearly indicates that patients with ICI-related myocarditis have a distinct cytokine profile compared with healthy donors. Indeed, patients with ICI-related myocarditis cluster homogeneously close together and separated from healthy donors. We have already shown that the activation of the IL-6/Th1 axis is an indicator of severe CS-refractory irAEs, such as ICI-related myocarditis,^2^ hemophagocytic lymphohistiocytosis^25^ and cholangiohepatitis,^24^ which could suggest that the IL-6/Th1 axis may be a possible common blood biomarker for CS-refractory irAEs. It is important to note that CS-refractory, infliximab-refractory, or MMF-refractory myocarditis responded very well to anti-IL6R therapy, which could further support the role of this therapy in the treatment of refractory myocarditis. The few published case series of myocarditis with EMB have described T-cell infiltration, mostly CD8+ T cells intermixed with CD4+ T cells, and CD68+ macrophages.^6 36^ ICI-related myocarditis is histologically characterized by a more lymphohistiocytic inflammatory infiltrate with an increased CD68:CD3 ratio and increased PD-L1+ macrophages and myocytes,^36^ and by the transcriptional involvement of CD8 and the IFN-γ pathway.^38^ Our patients displayed significant blood increases in the proportion of Th1, Th1/Th17, and Th17 helper memory CD4 T-cell populations, suggesting a possible pathogenic correlation between the predominant blood immune T-cell activation and the T-cell immune infiltrates reported on EMB. These immune-related phenotypical and functional determinations need to be further sequentially explored in order to identify robust predictive biomarkers specific to ICI-related myocarditis

Interestingly, our longitudinal profiling of the immune cells by global immune cell CyTOF panel with 40 markers reported for the first time a possible role of Th17 in ICI-related myocarditis, especially in patients 8 and 9, who had refractory myocarditis but responded to IL6R blockade therapy. Importantly, IL-6 induces the development of Th17 cells from naïve CD4+ T cells.^39^ The key role that this pathogenic cytokine plays in the differentiation of Th17 cells could suggest that there is a causal link between the increase in IL-6 and the involvement of Th17 cells. This observation is very important in the context of CS-refractory irAEs because a number of reports have postulated that Th17 cells may play a role in CS-refractory irAEs in patients who respond to anti-IL6 therapies without providing direct evidence. Thus, the possibility that Th17 could be a potential predictive biomarker of irAEs and resistance or low response to CS is plausible but should be confirmed in a large population with different types of CS-refractory irAEs. This case series of ICI-related myocarditis supports the use of anti-IL-6 therapy in CS-refractory myocarditis, as we have reported before.^2^

The high proportion of non-classical monocytes in four or five patients was also consistent with an inflammatory disease, and the significantly reduced levels of CD31, a checkpoint receptor for monocyte FcγR-mediated phagocytic activity, have been associated with acute coronary syndromes. These immune correlates provide increased confidence in the diagnosis of myocardial disease; however, ^68^Ga-DOTATOC was the only method that had a 100% sensitivity for the identification of ICI-related myocarditis.

We should also acknowledge some limitations in this original and retrospective pilot study. First, the number of patients included in the study was limited. Histological confirmation was available in only one patient and was negative, and only two patients had coronary angiography to exclude acute coronary syndrome despite the clinical presentation with chest pain and elevated troponins at a mean age above 70 years. It might be explained by the poor health status of those patients with metastatic disease and the strong suspicion of ICI-related myocarditis as the median time for the onset of myocarditis from ICI initiation was 2 months, in agreement with the current literature. In addition, all patients had a favorable evolution after initiation of an IS treatment. Similarly, CMR was not performed in all patients, and the imaging protocol to detect myocarditis in our institution was based on T2 mapping and LGE, whereas T1 mapping of the whole myocardium was not systematic. Performing a systematic T1 mapping might have resulted in a higher rate of abnormal CMR as suggested by the recent study of Thavendiranathan *et al*. Finally, the use of a control group with NET patients without abnormal uptake of the myocardium on visual analysis might not have been the ideal control, but it was in our opinion the best option. Indeed, except for the detection of myocarditis, the current recruitment for ^68^Ga-DOTATOC in our institution is patients with NET tumors, and those patients are not usually treated with ICI, which limits the possibility of a comparison to a group of patient undergoing ICI treatments and without clinical suspicion of myocarditis.

Nonetheless, our data indicate a high sensitivity of ^68^Ga-DOTATOC PET/CT for the detection of ICI-related myocarditis, though its specificity should be further explored in future studies. Indeed, using a control population of patients with cancer treated with ICI without clinical suspicion of ICI-related myocarditis or a direct comparison of EMB guided by the pathological uptake seen on ^68^Ga-DOTATOC PET/CT would help to validate its diagnostic accuracy. Furthermore, the main advantages of ^68^Ga-DOTATOC PET/CT for the detection of ICI-related myocarditis are the absence of physiological uptake of the myocardium and that no special several days’ preparation regimen is needed by contrast to ^18^F-FDG PET/CT well suited for emergency setting.^20^

## Conclusion

^68^Ga-DOTATOC PET/CT showed promising results as an imaging modality for the diagnosis of ICI-related myocarditis. Characterized by hs, it might be of value at the early stage of the disease in patients with suggestive clinical symptoms who may not yet present myocardial damage on CMR imaging. Its value is supported by a good concordance with serum levels of hs troponin I, the inflammatory IL-6/Th1 cytokine markers and immune correlates, including Th17. ^68^Ga-DOTATOC PET/CT was also useful for detecting concomitant myositis. These findings should be considered in light of certain limitations, including the smaller number of patients and the retrospective study design. Thus, the diagnostic value of ^68^Ga-DOTATOC PET/CT for the detection of ICI-related myocarditis should be confirmed in future studies including a larger population and histological documentation of myocarditis.

## Data Availability

Data are available upon reasonable request.
