# Correction: ^68^Ga-DOTATOC PET/CT to detect immune checkpoint inhibitor-related myocarditis

**DOI:** 10.1136/jitc-2021-003594corr1

**Published:** 2022-05-31

Boughdad S, Latifyan S, Fenwick C, *et al*. ^68^Ga-DOTATOC PET/CT to detect immune checkpoint inhibitor-related myocarditis. *J Immunother Cancer* 2021;9:e003594. doi: 10.1136/jitc-2021-003594.

This article has been corrected since it was first published online. The acknowledgements have been removed, as consent was not provided.

