# Supplementary material for: 68Ga-DOTATOC PET/CT to detect immune checkpoint inhibitor-related myocarditis
Source: J Immunother Cancer. 2021 Oct 21;9(10):e003594. doi: 10.1136/jitc-2021-003594 (PMC8543755; doi:10.1136/jitc-2021-003594)
Supplement: Supplementary data [file jitc-2021-003594supp001.pdf]

Supplemental figure S1, panel A Flow chart of patients with suspicion of ICI-related myocarditis as a function of their work up for ^{68}Ga-DOTATOC PET/CT (A).

Supplemental figure S1, panel b Delay between clinical suspicion of ICI-related myocarditis as a function of the time course of the evolution of hs Troponin I levels in patients that had ^{68}Ga-DOTATOC PET/CT.

Supplemental figure S2 Pie chart of the percentage of patients with elevated Troponin I, abnormal LVEF, abnormal ECG and abnormal NT-ProBNP among patients who underwent CMR imaging and those who received PET/CT ^{68}Ga-DOTATOC.

Supplemental figure S3 ROC analysis curve showing the comparison between MBRpeak calculated on the free wall of patients with ICI-related myocarditis (9 positive cases) and a control group of NET tumors patients (39 negative cases).

Supplemental figure S4 (A) Fused ^{68}Ga -DOTATOC PET/CT images in the coronal plane showing pathological uptake in the paravertebral and intercostal muscles in 5 patients: a (patient 3), b (patient 4), c (patient 6), d (patient 7), e (patient 9); SUV scale 0–2 g/mL.

Supplemental figure S4 (B) Fused ^{68}Ga -DOTATOC PET/CT images in the coronal plane showing pathological uptake in the paravertebral and intercostal muscles in 5 patients: a (patient 3), b (patient 4), c (patient 6), d (patient 7), e (patient 9); SUV scale 0–2 g/mL.(B)^{68}Ga -DOTATOC PET/CT images of a 75-year-old male (patient 9) with myocarditis and diffuse myositis (neck, intercostal, paravertebral muscles and pelvic area and a 65-year-old male (patient 11) without myositis; a/e: maximum intensity projection images; b/f: CT in the coronal plane; c/g: fused PET/CT image in the coronal plane; and d/h: PET images in the coronal plane; SUV scale 0–2 g/mL.

Supplemental figure S5 (A) ROC analysis curves were generated to evaluate AUC values, sensitivity, specificity, ppv, npv for cytokines of interest. (B)Principal component analysis (PCA) on cytokine markers.

Supplemental figure S6 ^{68}Ga-DOTATOC fused PET/CT images in the axial plane showing a panel patients with suspicion of ICI-related Myocarditis (a, b and c) and patients with NET tumors (d, e and f); SUV scale 0–2 g/mL.
