# Supplementary figures and images for: 68Ga-DOTATOC PET/CT to detect immune checkpoint inhibitor-related myocarditis
Source: J Immunother Cancer. 2021 Oct 21;9(10):e003594. doi: 10.1136/jitc-2021-003594 (PMC8543755; doi:10.1136/jitc-2021-003594)

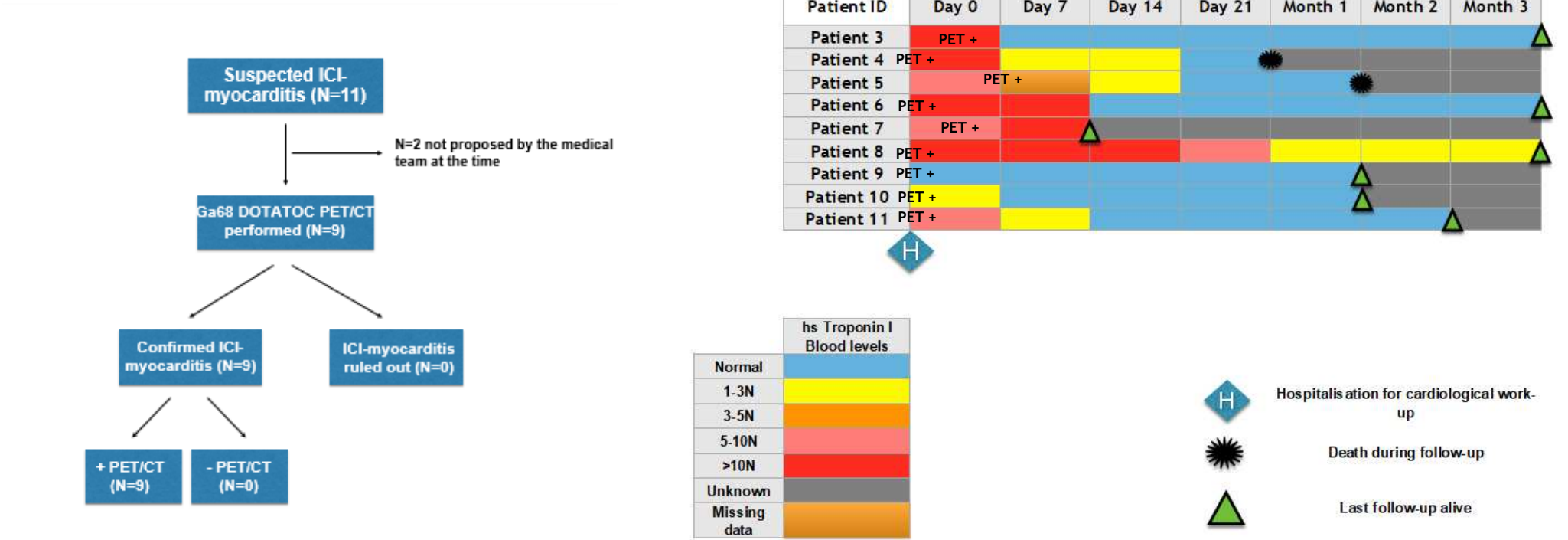

Supplemental Figure S1 a et S1b

Supplement: Supplementary data [file jitc-2021-003594supp002.pdf]

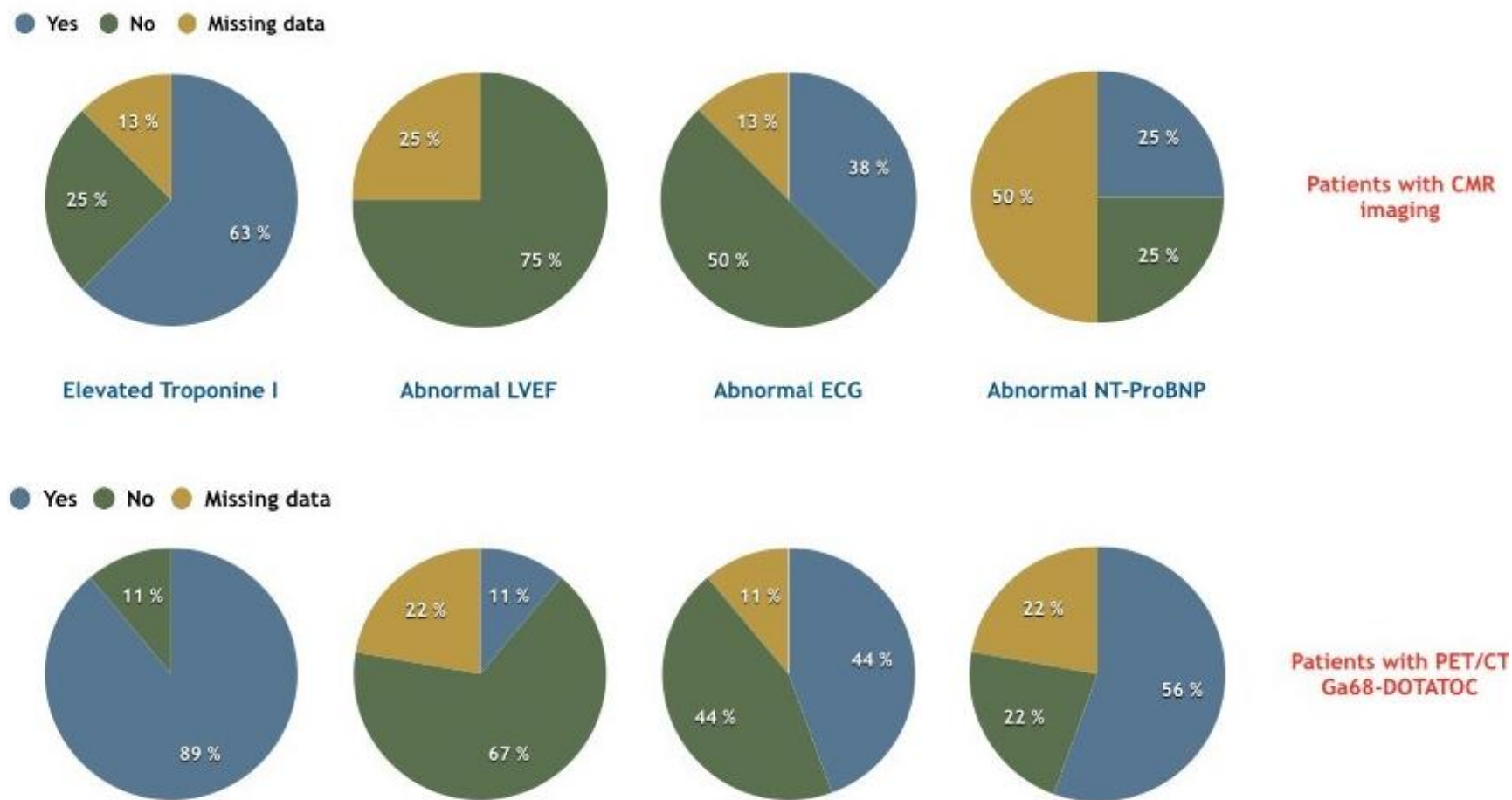**Figure S2**

Supplement: Supplementary data [file jitc-2021-003594supp003.pdf]

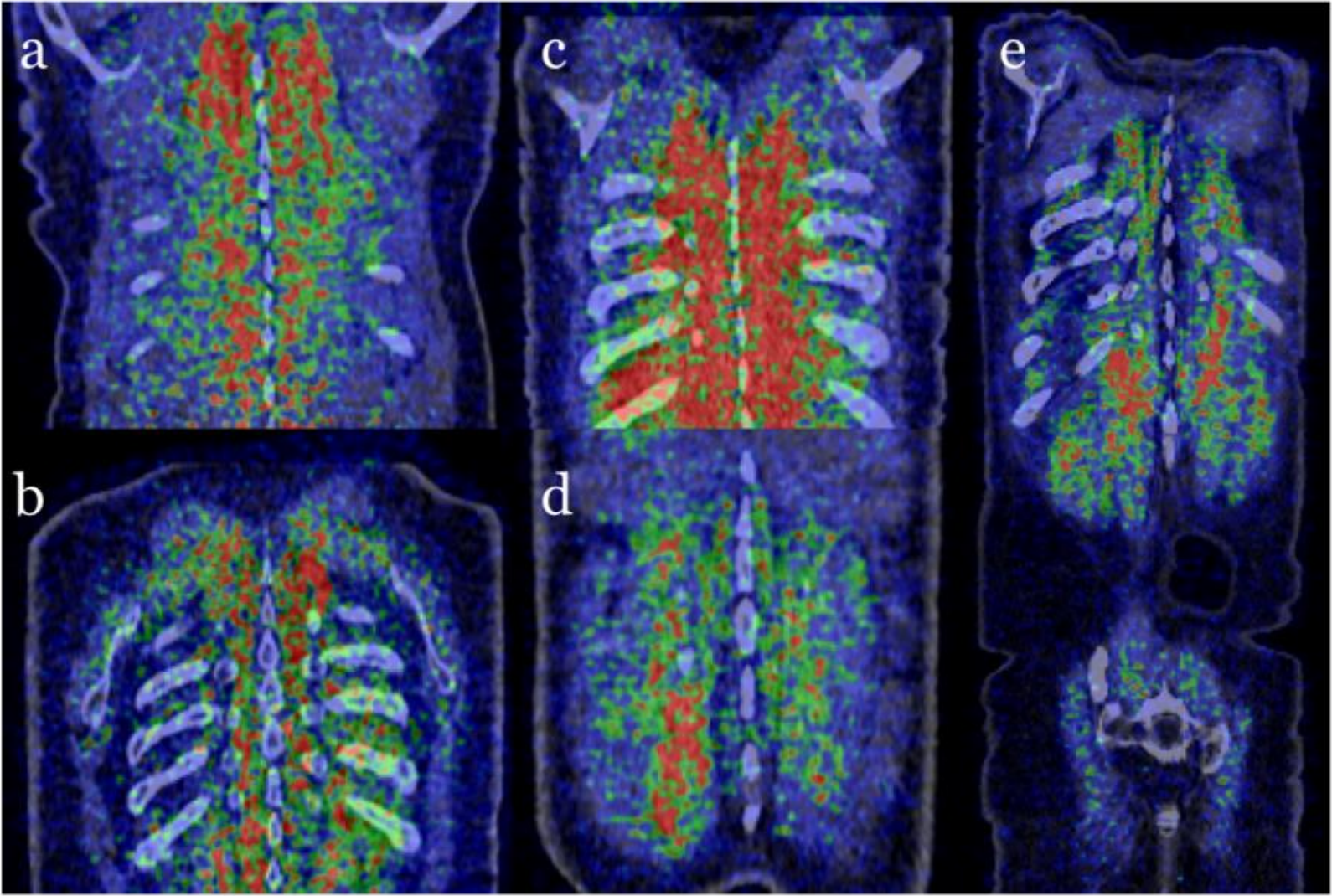

**Supplemental Figure S4 A**

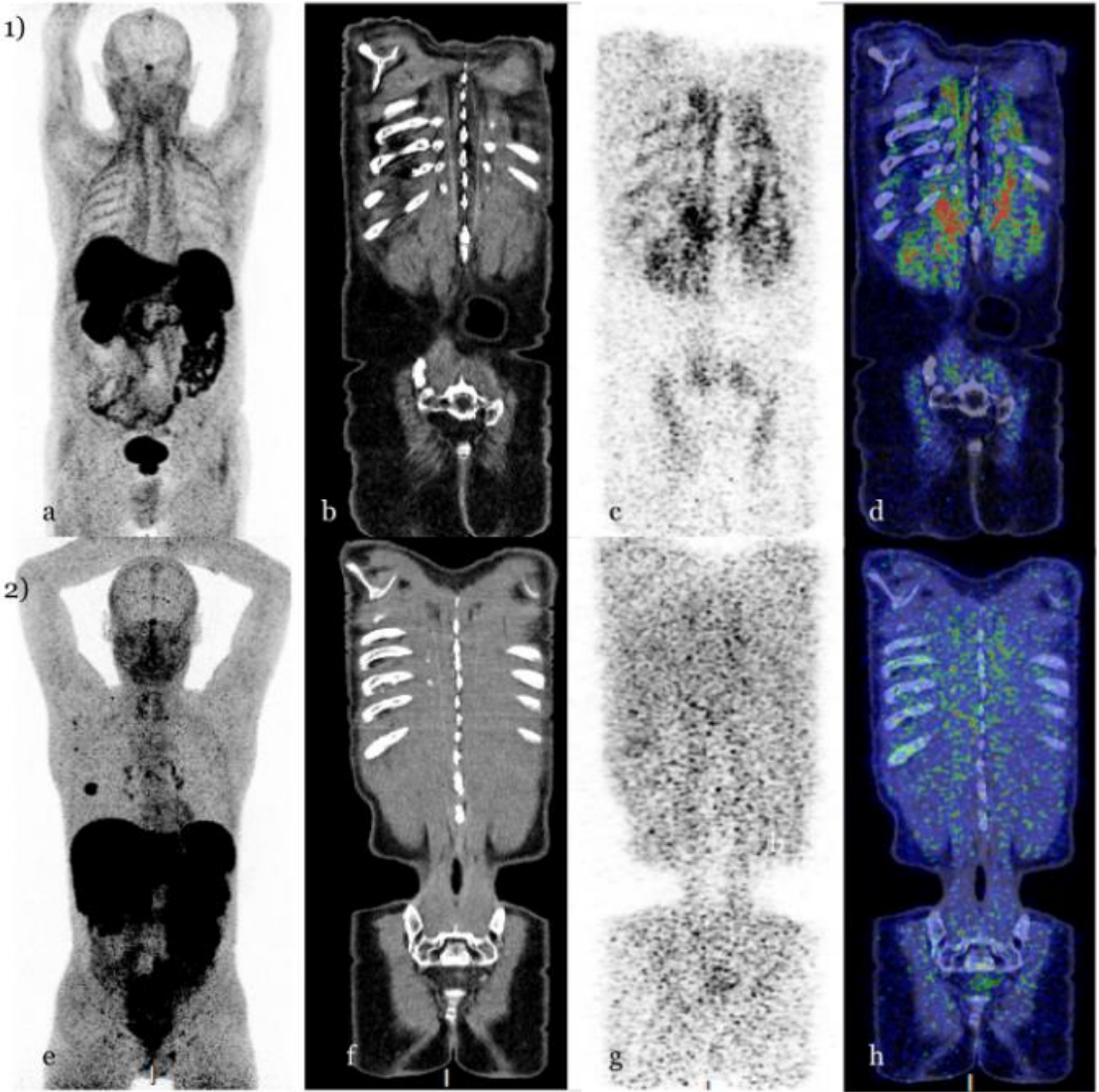

Supplemental Figure S4 B

Supplement: Supplementary data [file jitc-2021-003594supp005.pdf]

A

| Marker | Cut-off | sensitivity | specificity | ppv   | npv   | AUC   |
|--------|---------|-------------|-------------|-------|-------|-------|
| IL-6   | 12.75   | 1.000       | 0.714       | 0.996 | 1.000 | 0.786 |
| CXCL9  | 13.5    | 0.951       | 1.000       | 1.000 | 0.241 | 0.989 |
| CXCL10 | 22      | 0.956       | 1.000       | 1.000 | 0.259 | 0.990 |
| CXCL13 | 103.5   | 0.976       | 0.857       | 0.998 | 0.353 | 0.941 |

B

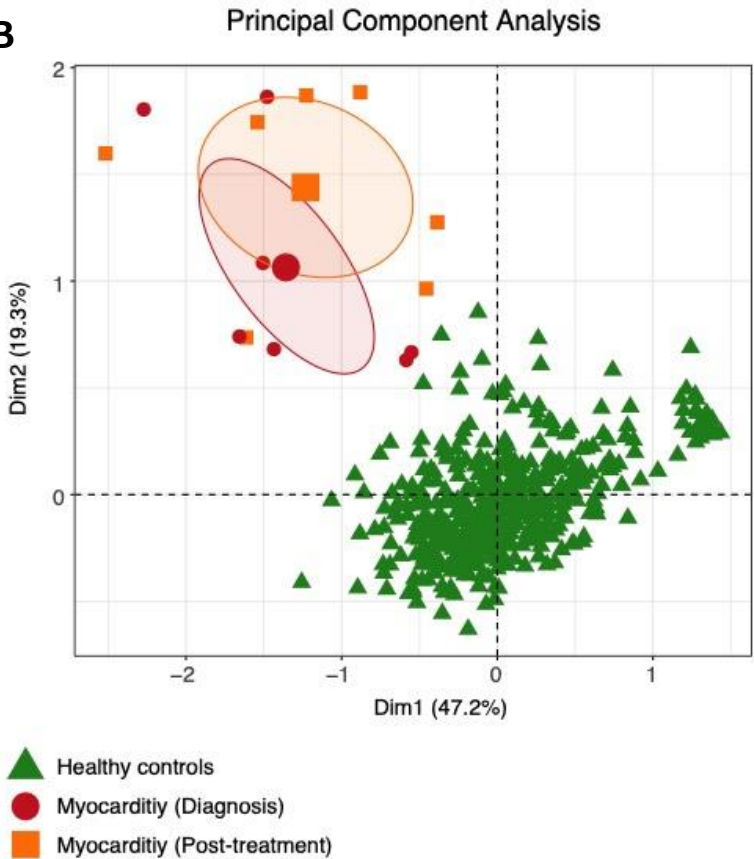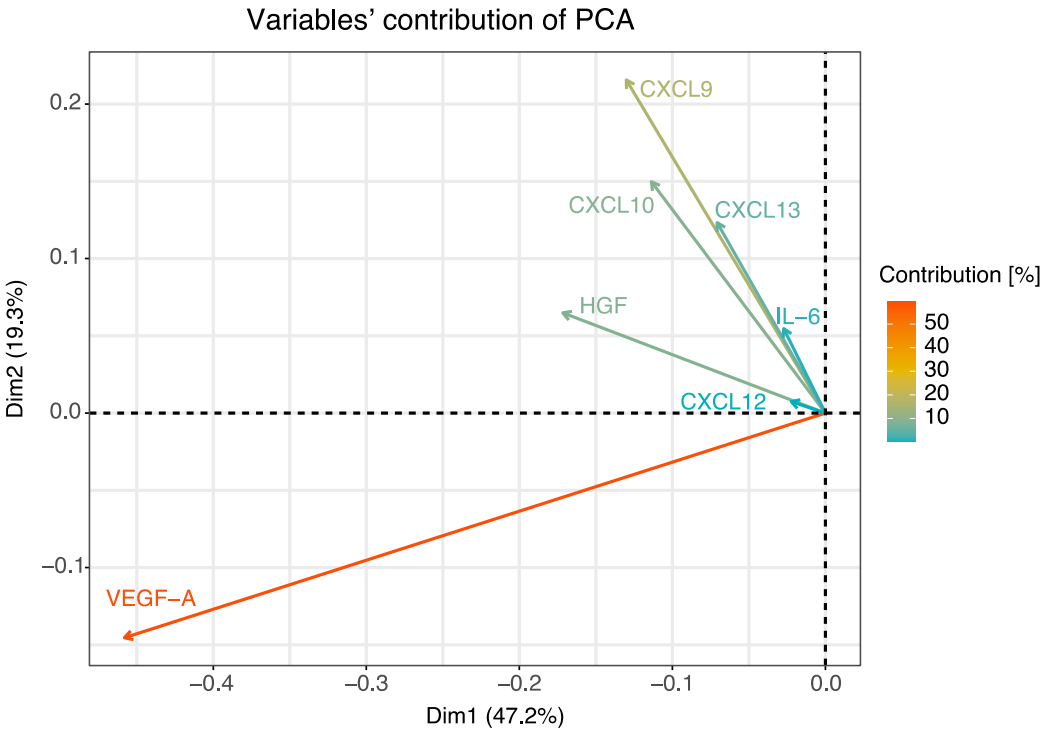

Figure S5

Supplement: Supplementary data [file jitc-2021-003594supp006.pdf]

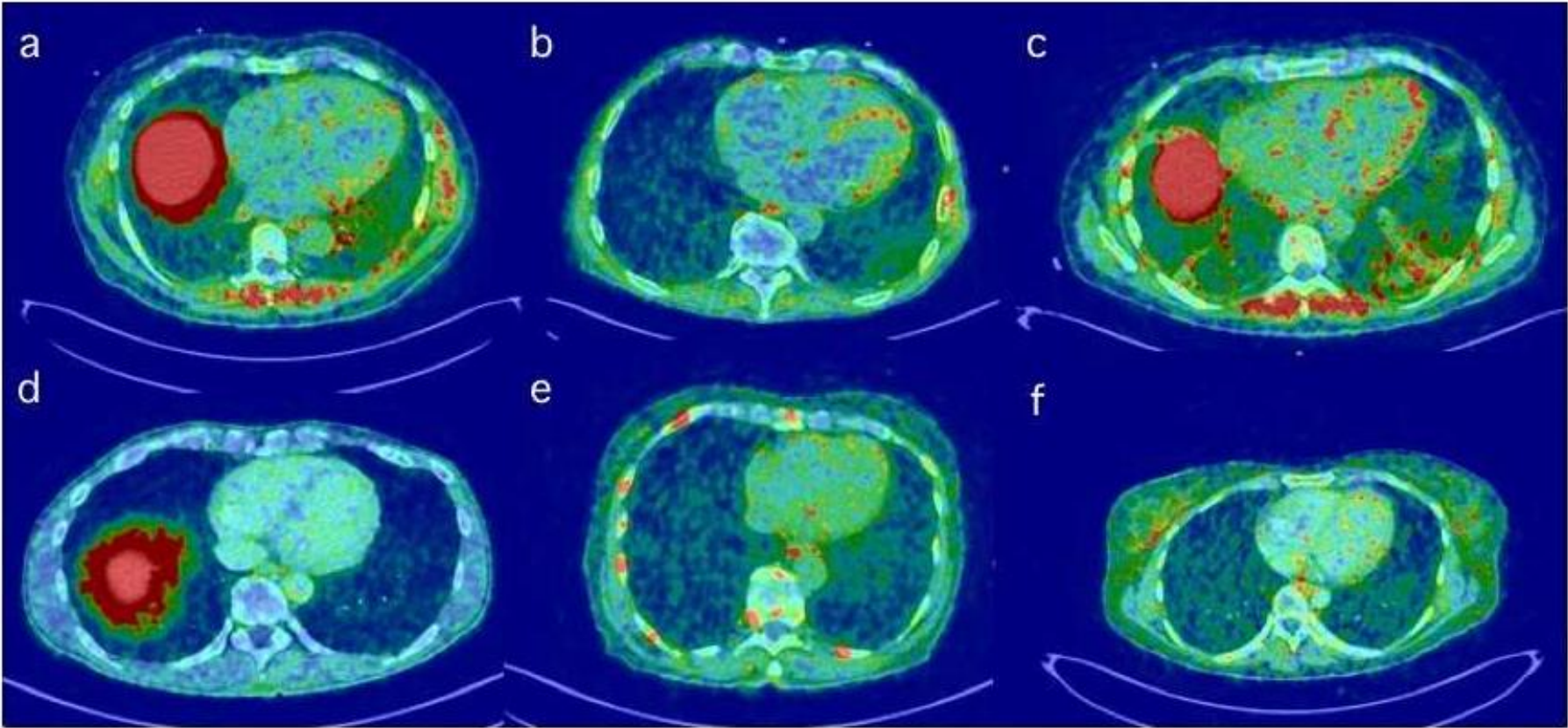

Supplemental Figure S6

Supplement: Supplementary data [file jitc-2021-003594supp007.pdf]
