# Supplementary material for: 68Ga-DOTATOC PET/CT to detect immune checkpoint inhibitor-related myocarditis
Source: J Immunother Cancer. 2021 Oct 21;9(10):e003594. doi: 10.1136/jitc-2021-003594 (PMC8543755; doi:10.1136/jitc-2021-003594)
Supplement: Supplementary data [file jitc-2021-003594supp004.pdf]

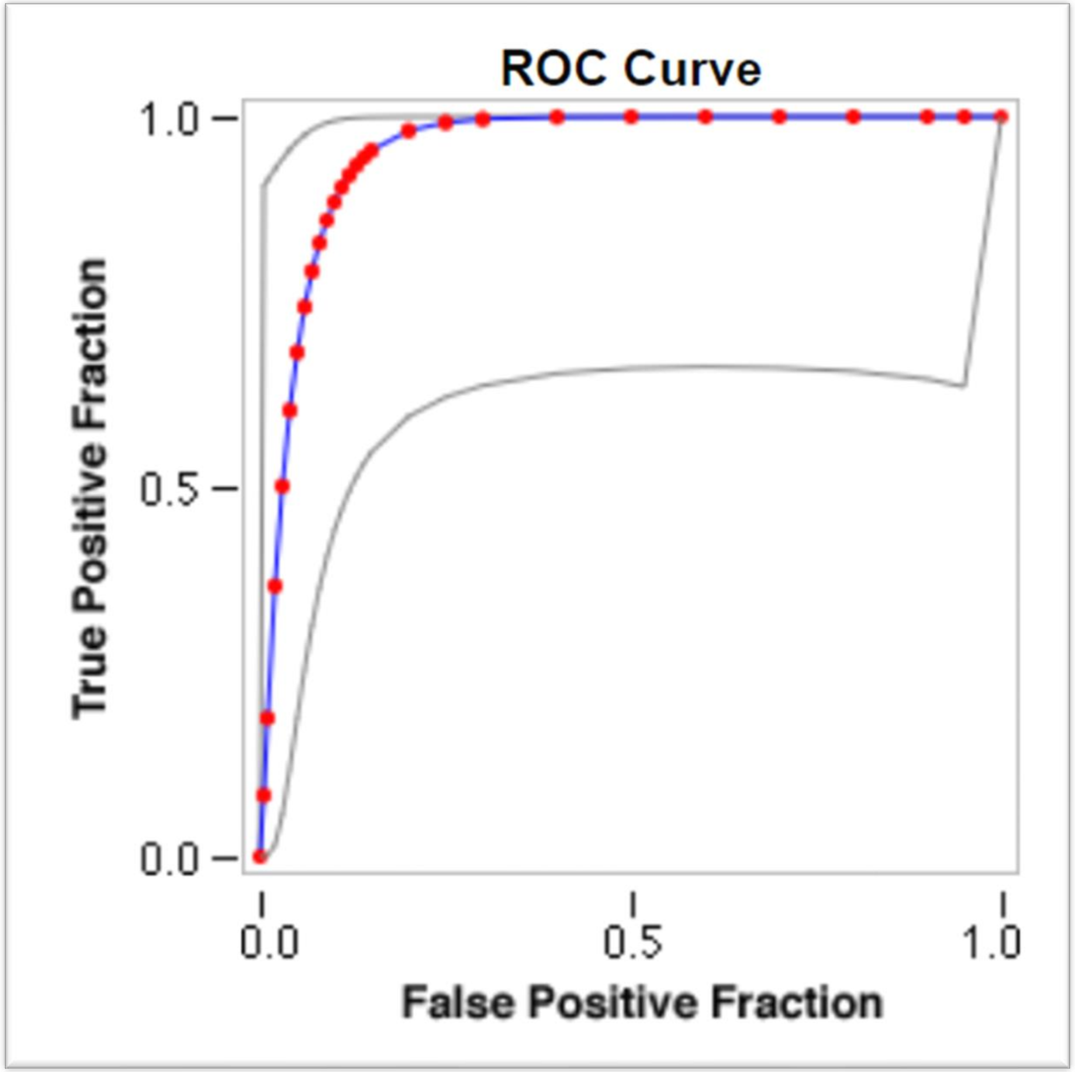

Summary statistics

- **Total cases: 48**
- **Positive cases: 9**
- **Negative cases: 39**

- **Fitted ROC Area: 0.954**

Supplemental Figure S3
